# Impact of Authentic Leadership on Nurses' Ethically Oriented Practices and Patient-Oriented Outcomes: A Multilevel Analysis

**DOI:** 10.1155/jonm/5336957

**Published:** 2025-03-30

**Authors:** Dina Metwally, Haroon Bakari, Mohamed Metwally

**Affiliations:** ^1^Helwan Business School, Helwan University, Cairo, Egypt; ^2^Southampton Malaysia Business School, University of Southampton Malaysia, Johor Bahru, Malaysia; ^3^Defence Studies Department, King's Centre for Military Ethics, Defence Academy of The United Kingdom, School of Security Studies, King's College London, London, UK; ^4^European Universities in Egypt, University of London Programmes (Academic Direction by London School of Economics), New Administrative Capital, Egypt

**Keywords:** authentic leadership, compassion at work, multilevel analysis, nursing ethics, patient-oriented performance

## Abstract

**Objectives:** Authentic leadership is considered to be a popular leadership style within the health sector. However, despite the team-oriented work context in hospitals, most research in the health sector is based on data collected from individuals and not teams. Utilizing a multilevel modeling approach, this study aimed to examine the impact of authentic leadership on nursing outcomes at both individual and team levels. It aims to explore the impact of team-level authentic leadership on nursing outcomes at both team and individual levels.

**Methods:** A survey was conducted in hospitals operating in Cairo, Egypt to test the proposed model. Data were collected from 40 nurse supervisors and 200 nurse subordinates in a time-lagged design. Given the multilevel nature of the data (individuals nested within groups), the study's hypotheses were tested utilizing multilevel modeling that incorporates the nonindependence of observations obtained from lower levels nested within higher levels.

**Results:** Results suggest that the hypothesized model was a good fit to the data (CMIN/DF = 1.362, CFI = 0.957, TLI = 0.955, IFI = 0.958, and RMSEA = 0.049). Empirical results suggest that team-level authentic leadership positively predicted nursing teams' performance in improving patient comfort (*β* = 2.17, *p* < 0.05) and their ethically oriented nursing practices (*β* = 0.664, *p* < 0.05). Nurses' ethically oriented practices positively predicted nurses' patient-oriented performance (*β* = 0.188, *p* < 0.05) and mediated the link between authentic leadership and patient-oriented performance (UCI-0.6744, LCI-0.0474). The relationship between authentic leadership and ethically oriented practices was moderated by nurses' compassion at work (*β* = 0.129, *p* < 0.05), such that it was stronger when nurses had higher levels of compassion.

**Conclusion:** Nursing supervisors who practice authentic leadership can enhance nursing teams' performance regarding patient comfort. Authentic leadership can also foster ethical orientation in nurses. Furthermore, a nurse's compassion at work has a synergetic effect with authentic leadership to increase ethically oriented practices.

## 1. Introduction

Nursing performance is a crucial factor in healthcare organizations and is influenced by physical, cognitive, and organizational factors [1]. Many of these factors are embedded in leadership and management strategies that foster motivation and support for nurses [2, 3]. Research underscores the pivotal role of nursing leadership in enhancing nurse performance and achieving organizational objectives [4]. One leadership style which is extensively discussed in healthcare literature is authentic leadership (AL) [5–11]. Recent studies highlight the significant impact of AL on nursing performance [9, 12, 13]. Authentic leaders promote openness in information sharing, uphold core and incremental values, spread positivity, and focus on fostering authenticity among their followers [14, 15]. In the nursing context, AL is associated with high-quality patient outcomes related to care, comfort, and compassion [7].

Few studies have focused on the set of activities related to patient care, specifically caring nurse patient interaction (CNPI) [16]. CNPI encompasses physical and clinical care as well as emotional and psychological support. It involves addressing patients' feelings and emotions and providing them with helpful information to facilitate the healing process and foster their mental well-being in hospitals [17]. Authentic leaders can enhance CNPI through their genuine and transparent behaviors by fostering a supportive environment which encourages nurses to engage deeply with their patients. This supportive environment promotes emotional and psychological care which are both essential components of CNPI. Therefore, the integration of AL and CNPI can lead to improved nurse performance, resulting in higher levels of patient comfort performance (PCP).

Nevertheless, nurses confront situations daily where they are bound to make ethically oriented decisions [18, 19]. Ethically oriented nursing practices (EONP) are crucial for maintaining the quality of care. Unfortunately, there has been a disproportionate focus on the technical aspects of nursing care, often neglecting the ethical dimensions [20]. Authentic leaders can cultivate an ethical climate within healthcare organizations by emphasizing genuine and transparent behaviors. This ethical climate encourages nurses to adhere to high standards of ethical practices, ensuring that patient care is technically proficient and morally sound.

Scholars suggest that compassion at work (CAW) is a complex phenomenon that requires further investigation across different clinical and cultural contexts [21]. Compassion is seen as an interactional process through which one sees, feels, makes sense of, and acts to mitigate others' suffering [22]. It is also described as an expression of selfless love, tenderness, care, and affection towards others [23]. In organizations where CAW is practiced, employees experience a sense of favor and care, enjoying higher levels of psychological safety. Employees experiencing CAW show greater levels of mindfulness, kindness, and empathy towards others [24]. It is suggested that employees' trust in their supervisors and positive expectations guide their behaviors. Therefore, in groups with higher levels of interaction and exchange, individuals experience greater levels of CAW and a sense of connectedness [25–27].

Although using a multilevel analysis to understand issues related to the health sector is crucial, as most problems involve different levels or contexts related to individuals, organizations, workplaces, or management [28], there is a notable shortage of nursing research employing this approach to understand the role of authentic leaders in motivating nurses and influencing their behaviors as individuals and as teams [29]. In addition, few studies have focused on the set of activities related to patient care, specifically CNPI [16]. These interactions are crucial for quality nursing care and involve both clinical and emotional support.

A multilevel analysis approach is needed to better understand how AL can enhance these interactions and improve both nursing performance and patient outcomes [30]. The major contribution of this research is to employ a multilevel mechanism to investigate the impact of AL on nursing performance and related outcomes at both team and individual levels. Specifically, the study aims to explore the impact of AL on nursing PCP at the team level, in addition to EONP and patient-oriented performance (POP) at the individual level.

Bringing together the team and individual levels is a significant contribution, as teamwork is the cornerstone of providing quality healthcare services and achieving patient satisfaction [31]. In addition, this study aims to investigate the moderating/mediating effects of CNPI and CAW, further contributing to understanding these dynamics. By addressing these aspects, the research aims to provide a deeper understanding of how AL can enhance nursing performance and ethical practices, ultimately leading to improved patient outcomes and a more supportive healthcare environment.

## 2. Theoretical Background and Hypothesis Development

### 2.1. AL, PCP, and CNPI

Leadership literature is proliferated with different leadership styles [32]. However, AL is considered to be a promising leadership style that has a greater potential to bring positive outcomes to healthcare organizations [5, 9]. AL has been defined as “a leadership style that fosters and utilizes both psychological capacities and ethical climate to enhance positive self-development among themselves and their followers by engaging in four key leader behaviors, namely, internalized moral perspectives, self-awareness, relational transparency, and balanced processing” [33]. The AL style focuses on relational quality [32] and reflects leaders' positive psychological capacities, transparency, strong integrity, and credibility [34]. The theory of AL proposes that authentic leaders enhance employees' performance by developing positive capacities in followers [35]. Most of the AL literature focuses on the role of followers in leader–follower relationship dynamics. Therefore, follower-oriented constructs, such as trust in leadership, employee empowerment, and positive interaction between leaders and followers, are used as mediators to examine the mechanism that translates the impact of AL on both team- and organizational-level outcomes [36].

Nevertheless, the role of AL in the development of positive employees and organizational outcomes is proven in most studies [37–39]. It is suggested that authentic leaders enhance followers' commitment and engagement in the work [40] and resultantly enhance their performance [9]. Research suggests a positive association between nurses” leadership style and nurses' productivity [41].

Most research on comfort has covered physiological dimensions and has ignored emotional and psychological aspects [42]. PCP reflects the satisfaction of patients' three basic needs: need for relief, need for ease, and need for transcendence [43]. Patients' comfort can be achieved when their specific comfort needs are fulfilled (relief) and they are at ease, which means they experience calmness and contentment (ease), and they achieve the state where they rise above pain or problem (transcendence) [43]. Scholars suggest the need for a more comprehensive approach that incorporates emotional and psychological aspects in the study of comfort, given that psychological factors can influence the perception of comfort [44]. PCP is also defined as a complex, pleasant, subjective, holistic, and dynamic experience of patients that further reflects the satisfaction of specific needs [45].

Meanwhile, a review of the literature reveals that comfort is considered an outcome of nursing care [46–48]. Furthermore, it is found that AL has a positive impact on nursing patient care [8, 49, 50]. This is because authentic leaders play a significant role in shaping nurses' perceptions of patient care [50]. Raso [51] emphasizes the importance of AL in achieving improvements in healthcare environments, and its positive impact on nurses and patients. In addition research in nursing contexts shows that authentic leaders increase nurses' job satisfaction, [52], voice behavior [53], empowerment [54], reduction of incivility [55], and ensure increased performance and reduced turnover intention [12]. In addition, Elkholy et al. [56]explain that AL is deemed a positive relational leadership style in which leaders empower their followers by fostering an ethical and professional climate that fosters positive behaviors.

Based on previous discussions, it is possible to hypothesize the following:

• H1: A supervisor's AL has a positive impact on nurses' PCP at the team level.

Interactions and exchanges are vital to promoting a caring interpersonal environment at the workplace. In nursing care, the quality of interaction between nurses and patients is an important aspect of nurses' professional goals and responsibilities [57].

CNPI reflects four caring facets: (1) Humanistic care, (2) clinical care, (3) comforting care, and (4) relational care [58]. Humanistic care reflects nurse behavior that treats patients as fellow human beings, providing them with effective support as required. Clinical care is related to providing treatment in a timely and efficient manner, and the ability to deal with technical equipment and readiness to respond to critical conditions. Comforting care reflects taking care of patients' privacy, hygiene, and medication. Lastly, relational care reflects building trustworthy relations with patients in a way that eases their sufferings and enables them to cope with the problems they face in their lives [58, 59].

CNPI is described as an essential aspect of holistic nursing care that encompasses clinical and psychological care provided by nurses in healthcare settings [17]. In addition, the theory of fundamental care proposed by Feo et al. [60] and Kitson [61] describes that the nursing profession is composed of physical and psychological dimensions. Therefore, nursing is not limited to only clinical aspects as positive emotional and interpersonal interaction between patients and nurses is essential to nursing's fundamental care. These interpersonal exchanges between patients and nurses are conceptualized as CNPI and include clinical practices, emotional interaction, and positive nonverbal interaction between nurses and patients [62].

Moreover, research suggests that CNPI has a positive impact on POP [17, 62–64]. Scholars have found that patients who receive better CNPI have lower chances of rehospitalization and feel better security and safety [16, 59, 64]. It is also suggested that the atmosphere of the hospital that promotes transparency, genuineness, and ethical and moral values will positively enhance CNPI [65]. Furthermore, research highlights the need for testing the role of the patient-oriented communication approaches in fostering quality of care in hospitals [66]. Accordingly, a prominent level of patient–nurse interaction is expected to be positively reflected in the strength of the relationship between AL and PCP. Thus, a high level of patient–nurse interaction is expected to strengthen the relationship between AL and PCP. This leads to the following hypothesis:

• H2: Caring nurse–patient interaction moderates the relationship between the supervisor's AL and the nurses' PCP at the team level.

### 2.2. AL and EONP

The concept of ethics of care (EOC) may be defined as an ethical logic that is inherited from the notion of having concern for others and the ability to develop mutual relationships with those who are suffering from some pain [67]. EONP is a set of nurses' attitudes and behaviors that adhere to established professional norms and ethical codes of nursing care [68]. Ethical nursing advocates for the provision of nursing care to everyone based on the nature of disease and diagnosis, regardless of patients' status [69]. However, unequal distribution of resources, polarized leadership practices, and lack of strict policies create some ethical dilemmas in nursing care that promote unethical practices [70–72]. Ethical dilemmas in nursing practices may arise out of conflict between professional responsibility and personal values [73].

Key issues in the implementation of ethical nursing practices include heavy workload, time constraints, staffing, and financial problems [74]. In addition to such contextual characteristics, leadership practices are important to promote ethical practices in nursing [72, 75, 76]. Though a major premise of AL theory is followers' moral development [77], moral reasoning of EOC and ethical perspective in AL theory is conceptually similar [77]. Nevertheless, the core process between leaders and followers is the transference of values from leaders to followers through enactment and reinforcement.

AL is referred to as a process composed of self-awareness, balanced processing, moral and ethical perspectives, and relational transparency [78, 79]. Furthermore, AL is grounded in leaders' capacities to develop effective environments and constructive relationships with others to promote positive behavior in followers. Leaders do so by displaying awareness of self and others, displaying genuineness in their thoughts and actions, willingness to seek others' opinions, and commitment toward personal and core values [80, 81].

The theory of AL can provide a greater understanding of how to develop, promote, and implement ethical nursing practices in healthcare. As AL is a malleable construct [80], the introduction of this type of leadership is expected to foster positive capacities in nurses, which will support the development of transparent, genuine, and ethically motivated practices in nurses [82]. This leads to the following hypothesis:

• H3: Supervisor's AL has a positive impact on ethically oriented nursing practices

### 2.3. AL, POP, and EONP

POP is an approach to nursing that considers patients as a core part of the nursing care process [83]. It emphasizes involving patients' opinions, needs, and requirements in designing and implementing nursing interventions [84]. Kennedy et al. [85] defined POP as a nurse behavior that accommodates patients' requests, keeping patients at the center stage of nursing performance, making patients happy, and “suggesting alternative solutions to their problems.” Scholars have described POP in multiple contexts related to healthcare providers and patients. However, relative ambiguity regarding a clear definition of patient-oriented care has been considered a barrier to its implementation [86]. POP refers to a comprehensive nursing approach in which the nurse recognizes patients' illnesses through the perspective of the patients and considers patients' specific needs and preferences [87].

The POP encompasses various other sub-concepts, which include patient-oriented access, patient-oriented communication, POP, patient-oriented diagnosis, and patient-oriented interviews [88]. Research suggests that the implementation of POP has resulted in multiple positive outcomes, such as a decrease in average hospitalization of patients, increases in patients' satisfaction, reduction in costs of care, and enhancement of treatment effectiveness [87].

Ethics is described as a system of moral standards or ethical guidelines that govern an individual's or an organization's behavior [89]. Nurses are the healthcare professionals in charge of providing patients with care based on moral dilemmas [90]. The ability to manage and resolve moral dilemmas is necessary for delivering superior nursing care [91]. In addition, the improvement of ethical decision-making in nursing results in higher-quality treatment and services which are founded on scientific knowledge, as well as best practices. Moreover, the inability to resolve moral issues may hurt nurses' abilities and the standard of nursing care [92]. One of the key determinants of the quality of health care is patient happiness [93].

The past two decades has seen a substantial expansion of the body of literature on person-centered nursing care which has been referred to as a “new ethic of care” ([94], p. 6). This person-centered care focuses on specific targets such as patients [95], clients, older people [96], patients with specific diseases such as cancer, and so on [97]. Research shows that specific behaviors of the nurses, such as listening, communicating, teaching, honoring values, attending to needs, and treating each patient as an individual, complement nursing qualities to provide POP [98]. Therefore, it would make sense that nurses have a moral obligation to incorporate these qualities and behaviors into their nursing practice to accomplish POP [83, 98–100].

Based on the previous discussion, it is possible to hypothesize the following:

• H4: Ethically oriented nursing practices have a positive impact on nurse's POP at the individual level.

EONP creates an environment that assures patients' well-being and adds to the nobility of the nursing profession [101]. Research indicates that leadership practices are important in promoting ethical practices in nursing [74]. Furthermore, it is suggested that leaders play a significant role in enhancing patient-oriented care [102].

Research suggests that AL can bring changes in nurses that ensure patient-oriented care [103]. AL is grounded in leaders' capacities to develop effective environments and constructive relationships with others to promote positive behavior in followers [104]. Considering and combining AL aspects related to awareness, transparency, openness, inclusiveness, ethicality, and morality, authentic leaders garner follower support in developing a caring environment in organizations that promote patients' well-being [105]. In this regard, the ethical climate created by authentic leaders encourages nurses to adhere to high standards of patient care, ensuring that patient care is morally sound and technically proficient.

Based on H3 and H4, it is suggested that EONP, influenced by authentic leaders, is reflected in improved POP [49]. This is hypothesized in the following:

• H5: Ethically oriented nursing practices mediate the relationship between the supervisor's AL and the nurses' POP.

### 2.4. AL, EONP, and CAW

CAW may be described as a disposition and a wish to display sympathy to others or be able to understand others' pains and suffering [106]. Although compassion is included in the concept of empathy, it requires deeper levels of understanding that incorporate other peoples' pains and suffering as a part of self [23, 107].

Dutton et al. [108] presented CAW in a relational perspective in which two parties, the sufferer and the focal actor, are involved. As per their model, when a sufferer experiences a suffering triggered by pain, focal actors using a sensemaking perspective first notice it, then feels emphatic concern about the suffering of the sufferer, and then acts in a way to relieve the suffering. Thus, CAW refers to an “interpersonal process that involves noticing, feeling, sensemaking, and acting to alleviate the suffering of another person within a work environment” [108]. Researchers and practitioners have given substantial attention to this process, exploring its benefits for both the person experiencing suffering, the provider of compassion, and third parties who witness or hear about compassionate actions at work [108].

The social exchange theory describes the nature of human behavior. The theory posits that individuals tend to reciprocate and repay what they receive from social actors [109]. In this regard, employees' trust in their supervisors and their positive expectations will guide their behaviors. Therefore, in nursing groups, where greater levels of interaction and exchange occur, individuals experience greater levels of CAW and a sense of connectedness [110].

CAW is part of a value system and ethical orientation that needs to be promoted to foster quality of care in the health sector [111]. CAW reflects the prevalent environment in healthcare organizations. A CAW environment may be referred to as a positive condition at the workplace that fosters decent work, guarantees staff well-being, including safety and health, and encourages high-quality nursing care, which consequently enhances individual motivation and organizational performance [112, 113]. In a compassionate environment, nurses devote more time to supporting the patients, identifying their needs, respecting them, and valuing them [114, 115].

Evidence suggests that nurses lacking CAW are involved in routine duties and fail to provide high-quality compassionate care characterized by emotional and moral support to the patients [116]. Meanwhile, EOC refers to having concern for others and the ability to develop mutual relationships with those who are suffering from some pain [67]. A high level of CAW will likely strengthen the relationship between the supervisor's AL and EONP, leading to the following hypothesis.

• H6: The nurses' compassion at work moderates the relationship between the supervisors' AL and ethically oriented nursing practices.

**Figure 1:**
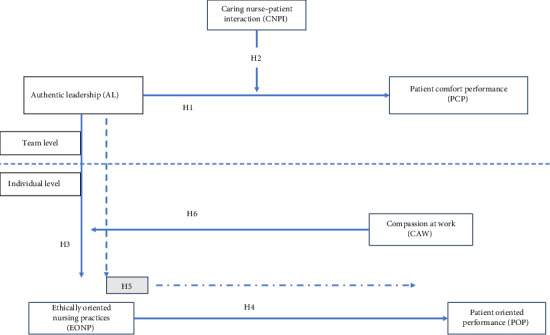
Research framework.

Figure 1 summarizes the research variables and hypothesized relationships. This framework depicts the multilevel nature of the study. At the team level, AL is modeled as a team-level predictor of nurses' performance related to patient comfort operationalized as PCP (Hypothesis 1). CNPI is employed as a moderator of this relationship between AL and PCP (Hypothesis 2). The third hypothesis reflects between-level relationships in which AL is hypothesized to impact EONP. Hypothesis 4 reflects the individual-level impact of ENOP on POP. Next, ENOP is modeled as a mediator between AL and POP (Hypothesis 5). Lastly, CAW is modeled as a moderator between team-level AL and individual-level ENOP.

In a nutshell, this study aims to investigate how AL at the team level influences nurses' performance in patient comfort (PCP) at the team level and EONP at the individual level. It also explores the moderating role of CNPI and CAW in these relationships. In addition, the study examines how EONP mediates the relationship between AL and POP at the individual level, ultimately seeking to understand the intricate interplay between these variables across different levels.

## 3. Research Methods

### 3.1. Methods

To test the proposed model, a survey was conducted in the hospitals operating in Cairo, Egypt. Initially, the general managers of 12 hospitals were contacted for their hospitals' participation in the study; however, only 7 have provided their consent. Before data collection, the questionnaire was translated using a back-translation procedure to ensure semantic equivalence of translation for the Arabic sample. Initially, a bilingual professional translated the questionnaire from English to Arabic and another professional then translated it back to English. After translation, pilot testing was conducted to ensure the appropriateness and clarity of the content.

Forty nursing teams working in 22 different departments, such as emergency, surgery, pediatrics, and internal medicine, were invited to participate in the survey. In total, the survey was administered to 40 supervisors and 200 subordinates. Some departments have multiple independent teams. The participants were briefed about the study's purpose and ensured that their responses would be kept confidential and used only for study purposes. In addition, as the procedure was a time-lagged design, the participants were asked to provide the initials of their names so that they could be easily approached the second time. Moreover, both the supervisors' and subordinates' questionnaires were coded to match the final responses.

Initially, supervisors were invited and were asked about their subordinates' details for data collection. Data collection was done in two stages. In the first stage, at the supervisors' level, the data were collected by distributing the questionnaire related to nurses' PCP and CNPI. While in stage 1, subordinates were contacted to provide data related to AL and CAW. In the second stage, data related to EONP and nurse POP were collected. Data collection took place between May 2021 and March 2022. The time difference between stage 1 and stage 2 was 5 months.

### 3.2. Measurements

#### 3.2.1. Authentic Leadership

AL was measured at the team level. A 16-item AL questionnaire was used to measure supervisors' AL style [33]. Nurses were asked to describe their direct supervisor on a five-point Likert scale (where 1 referred to strongly disagree and 5 referred to strongly agree). Examples of statements included “my direct supervisor … seeking feedback to improve interactions with others, saying exactly what he or she means, and listening carefully to different points of view before coming to conclusions”.

#### 3.2.2. Patient Comfort Performance

PCP was measured at the team level. It was measured using a three-item scale developed by Reference [58]. Supervisors were asked to rate each of the nurses they supervise on a five-point Likert scale (where 1 referred to strongly disagree and 5 to strongly agree). Statements included respecting patients' privacy (e.g., do not expose patients needlessly), taking patient's basic needs into account (sleeping, hygiene, etc.), and giving treatments or medication at the scheduled time.

#### 3.2.3. Caring Nurse Patient Interaction

CNPI was also measured at the team level. It was measured using a 23-item short scale developed by Reference [58]. CNPI was measured at the team level. This scale consisted of four dimensions: clinical care, relational care, humanistic care, and comforting care. Supervisors were asked to rate the behavior of the nurses they supervise using a five-point Likert scale. Examples of statements included knowing how to give the treatments (intravenous injections, bandages, etc.), helping patients to explore the meaning that they give to their health conditions, encouraging patients to be hopeful when it is appropriate, and respecting their privacy (e.g., do not expose them needlessly).

#### 3.2.4. Ethically Oriented Nursing Practices

EONP was measured at the individual level using the eight-item scale developed by Reference [68]. Nurses were asked to rate their level of agreement on a five-point Likert scale (where 1 referred to strongly disagree and 5 to strongly agree) about their work behaviors. Examples of the statements included statements such as “I tend to provide patient-centered nursing care”, “I usually communicate patients' needs to other health-care professionals”, and “I usually provide nursing care complying with related laws”.

#### 3.2.5. Patient-Oriented Performance

POP was measured at the individual level using four items adopted from Reference [85]. This scale was initially developed for customers. However, in this study, we replaced the word customers with patients. Nurses were asked to describe their work behaviors and attitudes using a five-point Likert scale. Examples of statements include how nurses felt about attending to the special requests of patients to the best of their ability, always fulfilling their work obligations with their patients in mind and working hard to please their patients.

#### 3.2.6. Compassion at Work

CAW was measured individually using a three-item scale developed by Lilius et al. [117]. Nurses were asked to express on a five-point Likert scale (where 1 = never, 7 = always) how frequently they experienced compassion (a) on the job, (b) from their supervisor, and (c) from their coworkers.

### 3.3. Analytical Strategy

Provided the multilevel nature of data (individuals nested within groups). The hypothesis of the study was tested utilizing multilevel modeling that incorporates the nonindependence of observations obtained from lower levels nested within higher levels [118–120]. In particular, we tested the mediation model with a level 2 predictor (AL), level 1 mediator (EONP), and level 1 outcome variable (POP). At level 2, moderation analysis was conducted with a level 2 moderator (CNPI) and level 2 outcome variable (PCP). For hypothesis testing, MLmed macro was utilized [121]. However, before testing the hypothesis, the intraclass correlation coefficient (ICC) was computed to analyze the degree of nonindependence among lower-level observations. According to Heck et al. [122], the value of ICC (0.05) is often regarded as the rough cutoff for the evidence of significant clustering. Nevertheless, even an insignificant amount of clustering where ICC is less than 0.05 may still have a significant effect on inferences. ICCs were computed for variables at lower levels, such as POP. The ICC was 0.206, suggesting the existence of variations between groups, which means multilevel analysis is warranted for hypothesis testing in this study [123, 124].

## 4. Results

### 4.1. Demographic Information

Regarding the number of teams included, eight teams were discarded because they did not submit usable data. Hence, the sample consists of 32 supervisors and 152 nurses nested under a supervisor. Details about the demographic characteristics of respondents are provided in Table 1.

### 4.2. Measurement Model

For the measurement model, confirmatory factor analysis (CFA) was conducted to ensure the reliability and validity of the constructs. Construct reliability refers to the consistency of the scores obtained by a scale over time and across multiple contexts. Construct reliability is measured at the item and construct levels: indicator reliability and internal consistency. Factor loadings were used as a criterion to establish the indicator reliability, that is, the extent to which an item contributes to the variance of the same construct. In a two-tailed test, the value of outer loadings surpassing 0.708 value with *t*-statistics greater than ±1.96 at a confidence interval of 5% will indicate an adequate level of indicator reliability [125, 126].

Internal consistency reliability is measured to indicate the extent to which all the items of a focal construct are associated with each other. Composite reliability is an estimate preferred over Cronbach Alpha. Hair et al. [127]suggested that the higher the CR values, the higher the reliability. As a cutoff value, they suggested that CR values between 0.90 and 0.95 are satisfactory, whereas above 0.95 will indicate the redundancy among items.

In the next step, convergent validity and discriminant validity are measured [128]. Convergent validity refers to the extent to which all the indicators of the same construct converge on the same construct, and the construct reflects the variance of all indicators. Construct validity is measured using the metric of average variance extracted. Values of AVE greater than 0.50 indicate satisfactory convergent validity. Discriminant validity measures the extent to which constructs of the model are distinct from each other and are tapping distinct concepts. For measuring discriminant validity, the values of Heterotrait-Monotrait Ratio (HTMT) are used [129].

The CFA results shown in Table 2 revealed that the model with six latent constructs, AL, CNPI, PCP, ENP, CP, and POP, had acceptable model fit indices as shown by (CMIN/DF = 1.362, CFI = 0.957, TLI = 0.955, IFI = 0.958, and RMSEA = 0.049). In addition, the standardized factor loadings for all items were greater than 0.50 [130]. Moreover, convergent and discriminant validity were established. For convergent validity, average variance extracted (AVE) and composite reliability (CR) were computed, and the results are provided in Table 3. The AVE for all the constructs was greater than 0.50 and ranged between 0.744 and 0.988. CR for all constructs was greater than 0.60 and ranged between 0.975 and 0.996, confirming convergent validity. Furthermore, the HTMT was computed for discriminant validity and the results are shown in Table 4. The recommended threshold for HTMT is less than 0.90. The HTMT between all constructs ranged between 0.012 and 0.145, confirming discriminant validity.

### 4.3. Convergent Validity

Average variance extracted (AVE), composite reliability (CR), and factor loadings were computed for convergent validity and the results are shown in Table 3.

### 4.4. Discriminant Validity

The HTMT was computed for discriminant validity and the results are shown in Table 4.

#### 4.4.1. Descriptive Statistics

Descriptive statistics such as mean, standard deviations, correlation, and reliability are provided in Table 5.

### 4.5. Hypothesis Testing

**Figure 2:**
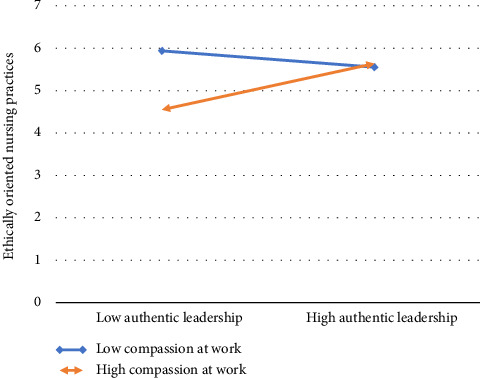
Interaction graph (authentic leadership∗compassion at work).

**Figure 3:**
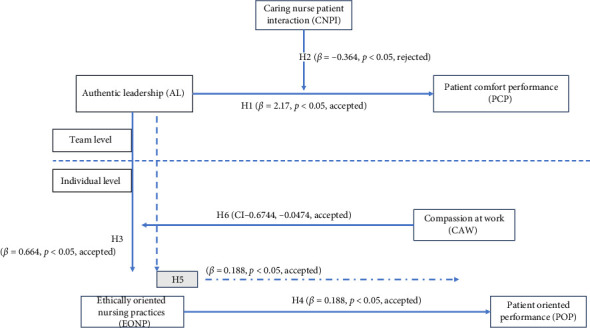
Summary of findings.

Hypothesis 1 stated that supervisor AL has a positive significant impact on the team's PCP. As delineated in Table 6, supervisor AL showed a positive effect on the team's PCP (*β* = 2.17, *p* < 0.05). Hypothesis 2 of the study stated that CNPI moderates the relationship between a supervisor's AL and team's PCP in a way in which the relationship will be strengthened when CNPI is high. The results in Table 6 revealed that though the coefficient is significant, it is in the opposite direction (*β* = −0.364,*p* < 0.05); hence, hypothesis 2 is not supported. Hypothesis 3 proposed that a supervisor's AL positively influences a nurse's EONP. The results in Table 7 revealed that the effects of AL are positive and significant on EONP (*β* = 0.664,*p* < 0.05). Hypothesis 4 stated that nurses' EONP has a positive impact on POP. The results in Table 7 showed that the effects of EONP on POP are positive and significant (*β* = 0.188, *p* < 0.05). Hypothesis 5 of the study stated that nurses' EONP mediates the relationship between supervisors' AL and nurses' POP. The results in Table 7 delineated that the indirect effect of a supervisor's AL on a nurse's POP through a nurse's EONP is significant and both lower level and upper level have the same sign and no zero is present between them (−0.6744, −0.0474), which represents that nurse's ethically oriented practices mediate the relationship. Lastly, hypothesis 6 stated that a nurse's CAW moderates the relationship between the supervisor's AL and the nurse's EONP, in a way in which the relationship will be strengthened when CAW is high and vice versa. The results in Table 6 provided support to the statement, as shown by the significant interaction term (*β* = 0.129, *p* < 0.05). Moreover, the interaction graph (Figure 2) revealed that nurse's EONP are high in the presence of more CAW; hence, hypothesis 6 is accepted. The summary of the findings is shown in Figure 3.

## 5. Conclusion and Discussion

This research is a significant contribution to nursing management and leadership literature. It has used a complex multilevel analysis to study AL at the team level and its impact on related outcomes, including PCP, EONP, and POP. While AL and PCP are measured at the team level, EONP and POP are measured at the individual level. The main contributions of this research are discussed below.

Firstly, the research has supported the positive impact of AL on PCP, both measured at the team level. This emphasizes the role of authentic leaders in motivating nurses to provide patient-oriented nursing care [50]. Nurses working with authentic leaders engage in caring, supportive, and extra-mile behaviors. PCP is achieved when nurses are dealt with fairly and transparently, which is a major characteristic of AL [131, 132].

Secondly, research findings have not supported the moderating impact of CNPI on the relationship between a supervisor's AL and PCP at the team level. Findings have indicated that CNPI does not impact the relationship between the supervisor's AL and PCP at the team level. Although CNPI is important in developing cordial relationships with patients and developing a sense of care and CAW [133], insignificant results of this research may be attributed to the presence of AL in the model which already incorporates balanced processing and relational transparency dimensions. These dimensions include authentic interactions and relational behaviors [134]. However, future research may explore this hypothesis further and see how CNPI can have a differential impact on PCP teams in the absence of AL in the model.

Thirdly, nurses confront situations daily where they are bound to make ethically oriented decisions [34]. This research has investigated the impact of a supervisor's AL on a nurse's EONP. Findings have supported the positive effects of supervisor's AL on nurses' EONP. Previous research has emphasized the role of authentic leaders in developing the moral and ethical levels of their followers [77, 135]. In the nursing context, the concept of EOC includes moral and ethical responsibilities toward patient care [67]. This finding supports other research findings that emphasize the role of AL in developing positive nursing behaviors in nurses [49, 136].

Fourth, this research investigated the relationship between EONP and nurses' POP. Findings have revealed the positive impact of EONP on nurse's POP. Research suggests that nurses' moral and ethical reasoning enhances the quality of care for patients in a healthcare setting [137]. Although empirical research on EONP and POP is scarce, theoretical and philosophical literature support the notion that ethics and morality are considered essential elements of nursing practices and are highly regarded for patient-oriented outcomes [137].

Fifth, the indirect relationship between the supervisor's AL and the nurse's POP through the mediating effect of EONP has also been investigated by this research. Research findings have suggested that AL influences nurses' POP not only directly (H4) but also significantly indirectly through the mediation of EONP. This finding is in line with previous research findings that support the role of AL in driving followers' positive behaviors [138–140].

Finally, the moderating impact of CAW on the relationship between AL and EONP has been examined. Findings have revealed that CAW strengthens the relationship between AL and EONP. CAW has been considered an important and core element of nurses' jobs [141]. Scholars and practitioners have considered CAW as an essential element of nurses' value system that enhances the ethical performance of nurses [138]. Employees experiencing CAW show greater levels of mindfulness, kindness, and empathy towards others [24]. Previous research has found that authentic leaders develop empathy and vitality in nurses [142]. Compassion reflects the quality of relationships among humans. AL, which is a positive leadership style, also stresses developing positive and genuine relationships with fellow human beings. The synergetic relationship between AL and CAW affecting EONP is an important insight of this study [143]. This study also answers the call for research by Stanley and Sebastine [144] who investigated compassion fatigue and compassion satisfaction in social workers and suggested testing AL and compassion in developing an ethical environment for workers.

## 6. Implications for Nursing Management

Our findings have several implications for nursing management. From the perspective of hospital-level decision-makers, it is essential to create an organizational climate that supports and facilitates AL practices by nurse managers. The dimensions of AL, including balanced processing, relationship transparency, self-awareness, and internalized moral perspective [1], should be used as a major criteria in the selection and training of nurse managers. These dimensions should also be employed as key evaluation criteria.

From the perspective of nursing managers, AL is considered a positive relational-leadership approach that empowers staff by creating a professional practice environment [56]. Nursing managers should be aware of the impact of their leadership practices on the performance of their nursing subordinates. They should promote EONP which are reflected in their subordinates' ethical performance. For example, the fair distribution of workload, working hours, and remuneration by nursing managers supports the creation of an ethical working environment [74]. In addition, nursing managers should foster a patient-centered working environment that enhances patient care and patient-centered performance.

The moderating impact of CAW on the relationship between AL and nursing subordinates' ethical performance has implications at both the hospital and management levels. At the hospital level, decision-makers are responsible for promoting a CAW environment that fosters decent work, guarantees staff well-being including safety and health, and encourages high-quality nursing care, which consequently enhances nursing and hospital performance [113].

In addition, nursing managers at various hospital levels are responsible for supporting this CAW environment. In this regard, nurses' trust in their managers and their positive expectations will guide their behaviors. If nurses believe their managers will fulfill their obligations, they will reciprocate positively. Compassionate nurses who work under the supervision of authentic leaders are more likely to demonstrate high levels of ethical performance. Accordingly, hospitals can enhance nursing performance, promote ethical practices, and improve patient outcomes, thereby creating a more supportive and effective healthcare environment.

## 7. Limitations and Future Recommendations

Although this research has employed multilevel modeling and has collected data from various sources, there may still be certain limitations. The first limitation, which poses a significant issue for determining causal relationships, is this study's cross-sectional design. Although recent methodological literature acknowledges the importance of cross-sectional design [145], future research can supplement this research by providing evidence through a longitudinal study. The current research model focuses on positive behaviors related to AL, but future research may incorporate other variables relevant to nursing practices. These variables could include work overload, nursing burnout, and coworker conflict. Such negative aspects of the work environment may have differential consequences for AL and nurse and patient outcomes [146].

## Figures and Tables

**Table 1:** Demographic characteristics of respondents.

Demographic characteristics	Frequency	Percent
Supervisors		
Gender		
Male	07	21.9%
Female	25	78.1%
Age		
26–31	03	9.4%
32–38	04	12.5%
39–47	17	53.1%
48–54	07	21.9%
Over 54 years	01	3.1%
Education		
Professional degree	24	75.0%
University degree	08	25.0%
Labor contract		
Permanent	24	75.0%
Temporary	08	25.0%
Tenure in team		
Less than 1 year	1	3.1%
1–2 Years	11	34.4%
3–4 Years	14	43.8%
5–6 Years	06	18.8%
Experience		
1–4 Years	04	12.5%
5–10 Years	20	62.5%
11–15 Years	03	9.4%
16–20 Years	05	15.6%
Tenure with supervisor		
1–2 Years	10	31.3%
3–4 Years	10	31.3%
5–6 Years	09	28.1%
7–8 Years	03	9.4%
Total	32	100%
Nurses		
Gender		
Male	35	23.0%
Female	117	77.0%
Age		
21–25	11	7.2%
26–31	43	28.3%
32–38	48	31.6%
39–47	34	22.4%
48–54	14	9.2%
Over 54 years	2	1.3%
Education		
Professional degree	102	67.1%
University degree	37	24.3%
University postgraduate	13	8.6%
Labor contract		
Permanent	83	54.6%
Temporary	69	45.4%
Tenure in team		
Less than 1 year	4	2.6%
1–2 Years	54	35.5%
3–4 Years	63	41.4%
5–6 Years	31	20.4%
Experience		
1–4 Years	27	17.8%
5–10 Years	87	57.2%
11–15 Years	18	11.8%
16–20 Years	20	13.2%
Tenure with current supervisor		
1–2 Years	55	36.2%
3–4 Years	48	31.6%
5–6 Years	39	25.7%
7–8 Years	10	6.6%
Total	152	100%

**Table 2:** Measurement model.

Model	CMIN	DF	CFI	TAG	IF	RMSEA
Baseline hypothesis model	2133.605	1567	0.957	0.955	0.958	0.049

**Table 3:** Factor loadings, composite reliability, and average variance extracted.

Variables	Loadings	CR	AVE
AL		0.979	0.745
AL1	0.710		
AL2	0.815		
AL3	0.804		
AL4	0.855		
AL5	0.898		
AL6	0.836		
AL7	0.868		
AL8	0.907		
AL9	0.847		
AL10	0.878		
AL11	0.870		
AL12	0.909		
AL13	0.905		
AL14	0.891		
AL15	0.891		
AL16	0.903		
CNPI		0.985	0.744
CNPI1	0.860		
CNPI2	0.861		
CNPI3	0.843		
CNPI4	0.867		
CNPI5	0.852		
CNPI6	0.869		
CNPI7	0.851		
CNPI8	0.871		
CNPI9	0.878		
CNPI10	0.871		
CNPI11	0.885		
CNPI12	0.870		
CNPI13	0.803		
CNPI14	0.850		
CNPI15	0.843		
CNPI16	0.857		
CNPI17	0.861		
CNPI18	0.856		
CNPI19	0.906		
CNPI20	0.846		
CNPI21	0.868		
CNPI22	0.894		
CNPI23	0.877		
PCP		0.975	0.906
PCP1	0.960		
PCP2	0.961		
PCP3	0.941		
PCP4	0.946		
EONP		992	0.938
ENP1	0.960		
ENP2	0.960		
ENP3	0.968		
ENP4	0.964		
ENP5	0.970		
ENP6	0.976		
ENP7	0.972		
ENP8	0.976		
CAW		0.996	0.988
CAW1	0.995		
CAW2	0.993		
CAW3	0.994		
POP		0.975	0.908
POP1	0.955		
POP2	0.960		
POP3	0.961		
POP4	0.936		

*Note: N* = 152.

Abbreviations: AL, authentic leadership; AVE, average variance extracted; CAW, compassion at work; CNPI, caring nurse–patient interactions; CR, composite reliability; EONP, ethically oriented nursing practices; PCP, patient comfort performance; POP, patient-oriented performance.

**Table 4:** Heterotrait-monotrait ratio (HTMT).

S.no	Variables	1	2	3	4	5	6
1	AL	1					
2	CNPI	0.128	1				
3	PCP	0.012	0.135	1			
4	EONP	0.117	0.024	0.033	1		
5	CAW	0.046	0.145	0.015	0.200	1	
6	POP	0.048	0.018	0.022	0.082	0.023	1

Abbreviations: AL, authentic leadership; CAW, compassion at work; CNPI, caring nurse–patient interactions; EONP, ethically oriented nursing practices; PCP, patient comfort performance; POP, patient-oriented performance.

**Table 5:** Descriptive statistics.

S.no	Variables	M	SD	1	2	3	4	5	6
1	AL	3.38	1.23	(0.979)					
2	CNPI	5.89	1.00	−0.13	(0.985)				
3	PCP	5.08	1.26	0.01	−0.13	(0.974)			
4	EONP	5.43	1.57	0.11	−0.02	−0.03	(0.992)		
5	CAW	4.90	2.32	0.04	0.14	−0.02	−0.20	(0.996)	
6	POP	3.12	1.33	−0.05	−0.01	0.02	−0.08	0.02	(0.975)

*Note: N* = 152. Reliabilities are presented in parenthesis.

Abbreviations: AL, authentic leadership; CAW, compassion at work; CNPI, caring nurse–patient interaction; EONP, ethically oriented nursing practices; M, mean; PCP, patient comfort performance; POP, patient-oriented performance; SD, standard deviation.

**Table 6:** Moderation of CAW and CNPI.

Variables	Ethically oriented nursing practices			Patient comfort performance		
	B	LL	UL	B	LL	UL
Intercept	7.761⁣^∗∗^	6.077	9.446	−4.302⁣^∗∗^	−8.056	−0.548
AL	−0.489⁣^∗^	−0.962	0.015	2.17⁣^∗∗^	1.217	3.139
CAW	−0.576⁣^∗∗^	−0.855	−0.267			
CNPI				1.55⁣^∗∗^	0.940	2.160
AL∗CAW	0.129⁣^∗∗^	0.043	0.214			
AL∗CNPI				−0.364⁣^∗∗^	−0.522	−206

*Note: N* = 152.

Abbreviations: AL, authentic leadership; AL∗CAW, authentic leadership∗compassion at work; AL∗CNPI, authentic leadership∗caring nurse–patient interaction; CAW, compassion at work; CNPI, caring nurse–patient interaction; LL, lower level; UL, upper level.

⁣^∗^*p* < 0.05.

⁣^∗∗^*p* < 0.01.

**Table 7:** Test of the mediation hypothesis.

	B	SE (standard error)	LL (lower level)	UL (upper level)
Outcome: EONP				
Within-effects				
Constant	3.193⁣^∗∗∗^	0.938	1.290	5.095
Between-effects				
AL	0.664⁣^∗^	0.275	0.105	1.222
Outcome: POP				
Within-effects				
Constant	5.704⁣^∗∗∗^	0.814	4.038	7.370
EONP	0.188⁣^∗^	0.076	0.035	0.340

**Between indirect effect**	**LL 95% CI**		**UL 95% CI**	

AL -> EONP -> POP	−0.6744		−0.0474	

*Note: N* = 152, *β* = coefficient. The upper and lower bounds are estimated with the Monte Carlo method.

Abbreviations: AL, authentic leadership; CAW, compassion at work; CI, confidence interval; CNPI, caring nurse–patient interactions; EONP, ethically oriented nursing practices; LL, lower level; PCP, patient comfort performance; POP, patient-oriented performance; SE, standard error; UL, upper level.

⁣^∗^*p* < 0.05.

⁣^∗∗∗^*p* < 0.001.

## Data Availability

Data are available upon request. Please contact the corresponding author.
